# Research trends and frontiers on antiphospholipid syndrome: A 10-year bibliometric analysis (2012–2021)

**DOI:** 10.3389/fphar.2022.1035229

**Published:** 2022-11-01

**Authors:** Teng Wu, Wei Huang, Jiaping Qi, Yixuan Li, Yuan Zhang, Huan Jiang, Jing Wang, Ju Zhang, Zhaoyu Jiang, Lin Chen, Zhenhua Ying

**Affiliations:** ^1^ The Second Clinical Medical College, Zhejiang Chinese Medical University, Hangzhou, China; ^2^ Zhejiang Provincial People’s Hospital, Hangzhou Medical College Affiliated People’s Hospital, Rheumatism and Immunity Research Institute, Hangzhou, China; ^3^ Qingdao University, Qingdao, China; ^4^ Bengbu Medical College, Bengbu, China

**Keywords:** antiphospholipid syndrome, bibliometric, CiteSpace, hotspots, VOSviewer

## Abstract

**Objectives:** A growing body of studies related to antiphospholipid syndrome (APS) have been published in recent years. Nevertheless, there is a lack of visualized and systematic analysis in the literature on APS. Hence, this study sought to conduct a bibliometric analysis to identify research status and discover frontiers in the field.

**Methods:** Articles and reviews concerning APS were acquired from the Web of Science Core Collection (WoSCC) database. CiteSpace, VOSviewer and a bibliometric online analysis platform were employed to conduct a visualization and knowledge-map analysis.

**Results:** A total of 1,390 publications regarding APS were identified. Globally, Italy contributed the most publications. The University of Padua was the most productive institution. Lupus ranked first in both the most published and most co-cited journals. Savino Sciascia and Spiros Miyakis were the most prolific and most co-cited authors, respectively. “Vitamin K antagonists (VKA)” and “immunoglobulin A (IgA)” were current research foci. Burst analysis of keywords suggested that “neutrophil extracellular trap (NET),” “direct oral anticoagulant (DOAC),” “open label,” “outcome,” “hydroxychloroquine (HCQ),” and “arterial thrombosis (AT)” were significant future research frontiers.

**Conclusion:** The scientific literature on APS has increased steadily in the past 10 years. The clinical studies on the treatment and mechanism research of APS are recognized as promising research hotspots in the domain of APS. The research status and trends of APS publications from the bibliometric perspective can provide a practical guide and important reference for subsequent studies by researchers and physicians in the domain.

## Introduction

Antiphospholipid syndrome (APS), also known as Hughes syndrome, is a systemic autoimmune disorder characterized by vascular (arterial, venous, microvascular) thrombosis and/or obstetric morbidity (Cohen and Isenberg, 2021). APS was first described by Professor Graham Hughes in 1983 (Hughes, 1983). It may occur alone, when it is called primary APS, or coexist with another autoimmune condition [mainly systemic lupus erythematosus (SLE)], that is, secondary APS (Luigi Meroni et al., 2019). The incidence and prevalence of APS are estimated to be approximately 2.1/100,000 per year and 50/100,000, respectively (Duarte-Garcia et al., 2019). The last 4 decades have witnessed a prominent evolution in the understanding of APS, and diagnostic methods have changed correspondingly. Although the classification criteria are frequently said to be unutilized for diagnosis, they are often applied to confirm the diagnosis in the domain of APS (Petri, 2020). The most famous seminars on the classification of APS were the conferences held at Sapporo in 1998 (Wilson et al., 1999) and Sydney in 2004 (Miyakis et al., 2006), which eventually reached an international consensus on the APS classification standard. The classification criteria for APS based on the Sydney standard are met when at least one clinical criterion (thrombosis or pregnancy morbidity) and at least one laboratory criterion [lupus anticoagulant (LA), anticardiolipin (aCL), or anti-beta two glycoprotein I (aβ2GPI) antibodies] are present. The above three antibodies are also collectively referred to as antiphospholipid antibodies (aPL). It was reported that the prevalence of aPL in the population was approximately 1–5%, but only a small proportion would develop APS (Cervera, 2017).

Of note, the aPL have recently been discovered in a considerable number of individuals with acute coronavirus disease 2019 (COVID-19), particularly in severe patients (Zhang et al., 2020; Karahan et al., 2022; Trahtemberg et al., 2021; Borghi et al., 2020), but it is still controversial whether they play a direct role in thrombosis or their existence is only a symptom of the disease’s main infectious proinflammatory state (Foret et al., 2021). A more clinically challenging situation is the presence of catastrophic APS (CAPS), which is characterized by rapidly progressive small vessel thrombosis in multisystem organs within 1 week. Although CAPS is extremely infrequent, developing in less than 1% of individuals with APS (Rodriguez-Pinto et al., 2018), treatment must be started immediately to combat a mortality rate of up to 30% (Cervera et al., 2018). The pathophysiology of CAPS remains unclear; however, it has been hypothesized that several triggering factors, such as infection, surgery, trauma, and malignancy, may cause endothelial injury, which leads to an overproduction of cytokines and a thrombotic storm in the microcirculation (Rodriguez-Pinto et al., 2016).

A substantial number of research on APS has been conducted over the last 10 years. Nevertheless, the continuously expanding amount of studies makes it challenging for scholars to maintain pace with the most recent findings. Although some literature reviews and meta-analyses can present summary findings, these means usually fail to achieve the capture of changing trends in publications, the evaluation of research contributions, and the prediction of research hotspots. Bibliometric analysis is a method that employs statistical and mathematical techniques to analyze scientific literature both qualitatively and quantitatively, which is used to detect research status and hotspots in a specific area (Lin et al., 2022). Moreover, bibliometrics can use relevant parameters to present contributions from different countries, institutions, and authors to help with subsequent experimental strategies and funding decisions (Deng et al., 2022a). Bibliometric analysis has been broadly used in the medical area (Aksoy et al., 2021; Ma et al., 2021; Wu et al., 2021; Zhu et al., 2021; Shen et al., 2022). However, no targeted bibliometric analysis of scientific literature on APS has been carried out to date. Therefore, this study sought to present a bibliometric analysis of APS literature published during 2012–2021, thereby depicting the current research status, identifying the hotspots and development trends, and providing new references for future research directions of APS.

## Materials and methods

### Data collection

Web of Science (WoS) is a broad and reputable database channel for obtaining international academic sources, including more than 12,000 international academic periodicals (Wu et al., 2021). Publications regarding APS were extracted from the Science Citation Index Expanded (SCI-Expanded) of the WoS Core Collection (WoSCC) and downloaded within 1 day on 24 May 2022. To ensure high relevance of the content, terms referring to “antiphospholipid syndrome” were searched by title (TI).

**FIGURE 1:**
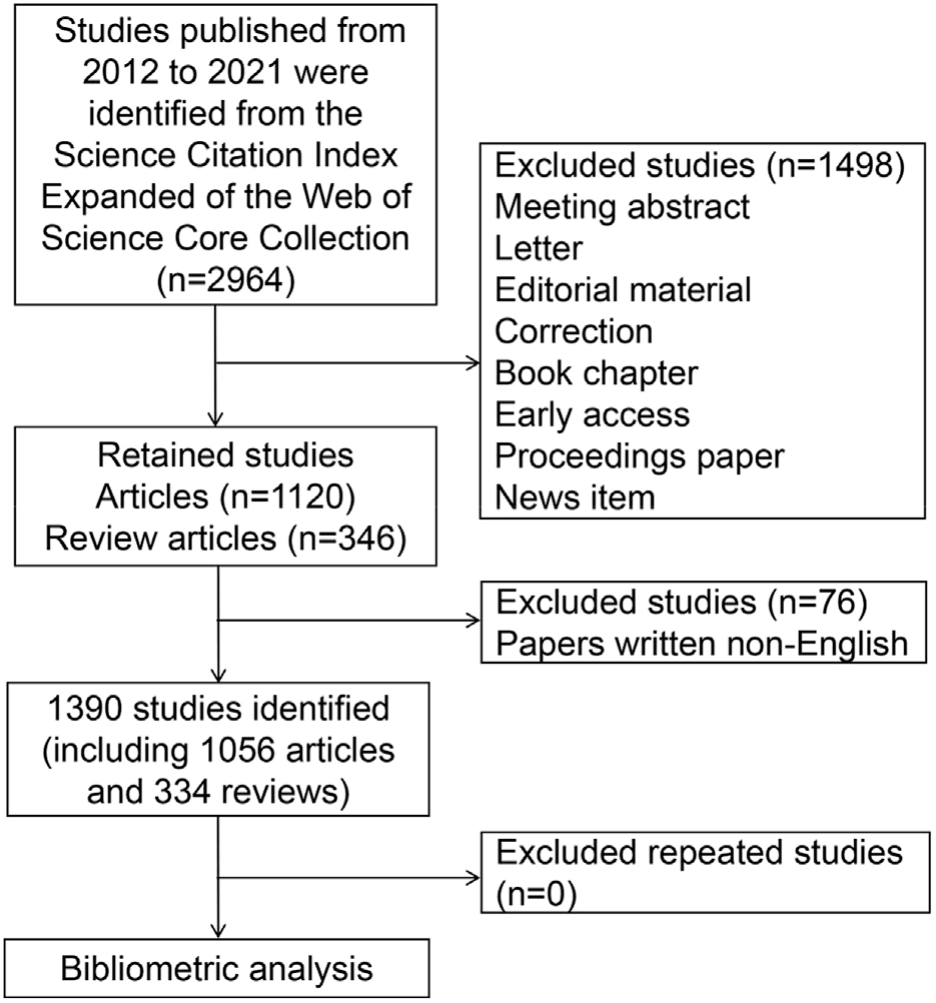
Flowchart of study retrieval and selection.

The retrieval formula was as follows: TI = (antiphospholipid syndrome OR anti phospholipid syndrome OR anti-phospholipid syndrome OR antiphospholipid antibody syndrome OR anti phospholipid antibody syndrome OR anti-phospholipid antibody syndrome OR Hughes syndrome). The time range was limited from 2012 to 2021. Only English-language articles and reviews were included in this study. A total of 1,390 documents were ultimately acquired and stored as “plain text.” Afterward, since CiteSpace can identify only files with a given name, these files were named “download_.txt”. Finally, these publications were imported into CiteSpace software for de-duplication (Figure 1).

### Data analysis

Microsoft Excel 2019 was used to evaluate the count of annual publications with APS research *via* a line chart. In addition, bibliometric analyses were carried out by three bibliometric tools. CiteSpace (version 5.8. R3), a Java-based tool, is a useful bibliometric software for analyzing scientific publications and presenting the knowledge framework *via* visualization results. Knowledge maps can intuitively capture the research hotspots and predict development trends within a specific area (Ma et al., 2021). In this work, the cooperation analysis of institutions and authors was conducted *via* CiteSpace. In addition, it was applied to implement a co-citation analysis of references and detect the burst references and keywords. In the network maps, each circle represents a research object, and the size of the circle is proportional to the publication or citation count. The line connecting the circles indicates the co-authorship or co-cited relationship, with thicker lines representing stronger collaboration or relevancy. The color of the line represents the time of the first co-authorship or occurrence, with a more yellow color meaning closer to 2021 and a more red color meaning closer to 2012. Centrality is an index used to evaluate the importance of elements in a map. The range of centrality is from 0 to 1. The higher centrality of an element means more frequent cooperation with other elements. Elements with a centrality value >0.1 usually imply significant influence, and the outermost ring of the element is displayed in purple. The following were CiteSpace’s arguments: timespan: 2012–2021 (slice length = 1), selection criteria: g-index (k = 25), pruning: pathfinder, pruning sliced networks, pruning the merged network.

VOSviewer (version 1.6.17), another practical bibliometric tool created by Van Eck and Waltman, can construct and visualize bibliometric networks, allowing for a better comprehension of the framework and evolutionary trajectory of scientific research (van Eck and Waltman, 2010). Moreover, VOSviewer can offer three various types of maps: network, overlay, and density visualization maps. In the present research, this application was used to perform a co-occurrence analysis of the keywords and citation and co-citation relationships of journals.

Furthermore, the co-authorship of countries was conducted using a bibliometric online analysis platform (https://bibliometric.com/).

## Results

### Analysis of publication trends

**FIGURE 2:**
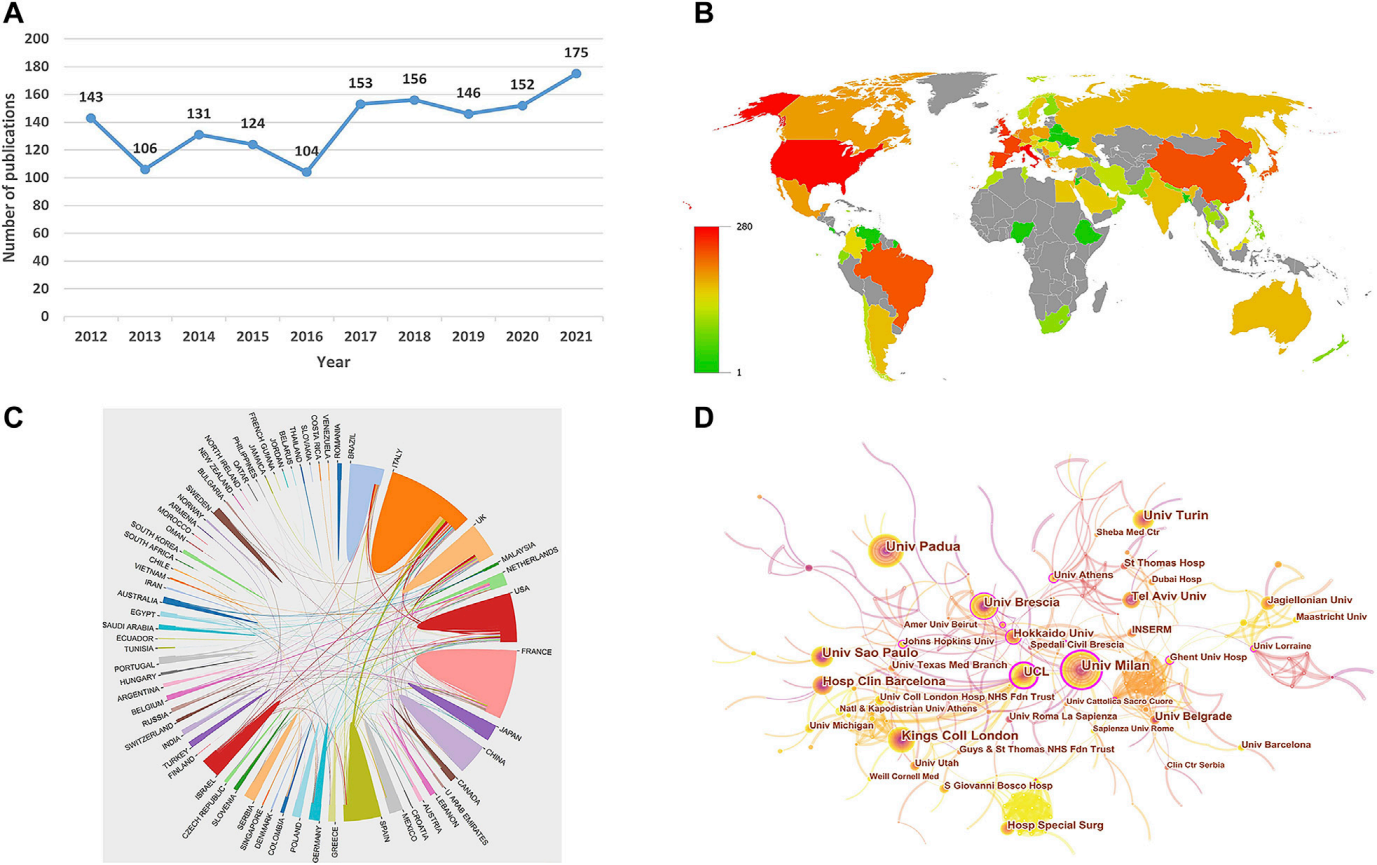
**(A)** Annual outputs of antiphospholipid syndrome (APS) from 2012 to 2021. **(B)** Map distribution on the sources of APS publications. **(C)** International cooperation analysis among countries by an online analysis platform. The curve between countries reflects the closeness of cooperation, thicker lines indicate closer cooperation. **(D)** International cooperation analysis among institutions by CiteSpace. Each circle represents an institution, and the size of the circle is proportional to the publication count. The circles with higher centrality (>0.1) are displayed with purple rings. The lines represent the strength of the co-authorship relationship, with thicker lines representing stronger collaboration. The color of the line represents the time of the first co-authorship, with a more yellow color meaning closer to 2021 and a more red color meaning closer to 2012.

After removing duplicates by CiteSpace software, a total of 1,390 publications of APS (1,056 articles and 334 reviews) were obtained from SCI-Expanded of WoSCC. Figure 2A exhibits the distribution of annual publications of APS literature, and research trends can be divided into two periods. From 2012 to 2016, the production of publications showed a gradual declining trend and reached a low point of 104 in 2016. From 2016 to 2021, the output of documents presented a rapid growth trend, with a peak in 2021. The quantity of papers published in 2021 was 1.68 times that in 2016.

### A bibliometric online analysis platform for visualization of country collaboration

Figure 2B shows a world map revealing the production of each country. A total of 1,713 institutions from 73 countries were involved in 1,390 documents. The top 10 countries on APS are displayed in Table 1. The top three countries with the most publications were Italy (273, 19.64%), the United States (258, 18.56%), and the United Kingdom (155, 11.15%). In addition, considering the influence of the demographic profile of various countries on the quantity of papers published, a ratio indicator of the number of articles published per million population was adopted. After adjusting for population size, Israel ranked first with 7.27 articles per million people. In terms of centrality, the top three countries were France (0.12), the United Kingdom (0.10) and the United States (0.09). The cooperation relationships among the countries are shown in Figure 2C. The width of the line represents the frequency of collaboration between two countries, and thicker lines indicate closer cooperation. As illustrated in Figure 2C, Italy collaborates closely with the United Kingdom, Spain, and the United States.

**TABLE 1:** Top 10 countries with the highest productivity related to antiphospholipid syndrome (APS).

Rank	Country	Count (% of 1,390)	Number of papers per million people	Centrality
1	Italy	273 (19.64)	4.59	0.07
2	United States	258 (18.56)	0.78	0.09
3	United Kingdom	155 (11.15)	2.31	0.10
4	Spain	135 (9.71)	2.85	0.05
5	France	130 (9.35)	1.93	0.12
6	China	115 (8.27)	0.08	0.07
7	Brazil	104 (7.48)	0.49	0.02
8	Japan	78 (5.61)	0.62	0.02
9	Israel	67 (4.82)	7.27	0.06
10	Netherlands	55 (3.96)	3.15	0.04

*Rank: based on the publication count. The demographic data were downloaded from the World Bank official website* (https://data.worldbank.org.cn/).

### CiteSpace for visualization of institution collaboration

Among the top 10 most productive institutions (Table 2), four were located in Italy, two in the United Kingdom, and one each in Brazil, Spain, Israel, and Serbia. Table 2 lists that the University of Padua contributes the largest number of documents (69, 4.96%), followed by the University of Milan (62, 4.46%), the University of Sao Paulo (53, 3.81%), the University of Turin (52, 3.74%), and King’s College London (50, 3.60%). As exhibited in Figure 2D, each node denotes an institution, and the size of the node signifies the amount of papers produced by the institution. With respect to centrality, the University of Milan and the University College London ranked first with 0.27, followed by the University of Brescia (0.14) and the University of Belgrade (0.09). Figure 2D demonstrates that the research on APS in Italy and the United Kingdom is led by the University of Padua and King’s College London, respectively.

**TABLE 2:** Top 10 most productive institutions related to APS.

Rank	Institution	Count (% of 1,390)	Centrality
1	Univ Padua (Italy)	69 (4.96)	0.03
2	Univ Milan (Italy)	62 (4.46)	0.27
3	Univ Sao Paulo (Brazil)	53 (3.81)	0.04
4	Univ Turin (Italy)	52 (3.74)	0.02
5	King’s Coll London (United Kingdom)	50 (3.60)	0.05
6	UCL (United Kingdom)	49 (3.53)	0.27
7	Univ Brescia (Italy)	47 (3.38)	0.14
8	Hosp Clin Barcelona (Spain)	42 (3.02)	0.04
9	Tel Aviv Univ (Israel)	39 (2.81)	0.03
10	Univ Belgrade (Serbia)	28 (2.01)	0.09

*Rank: based on the publication count.*

### CiteSpace for visualization of authors and co-cited authors

**FIGURE 3:**
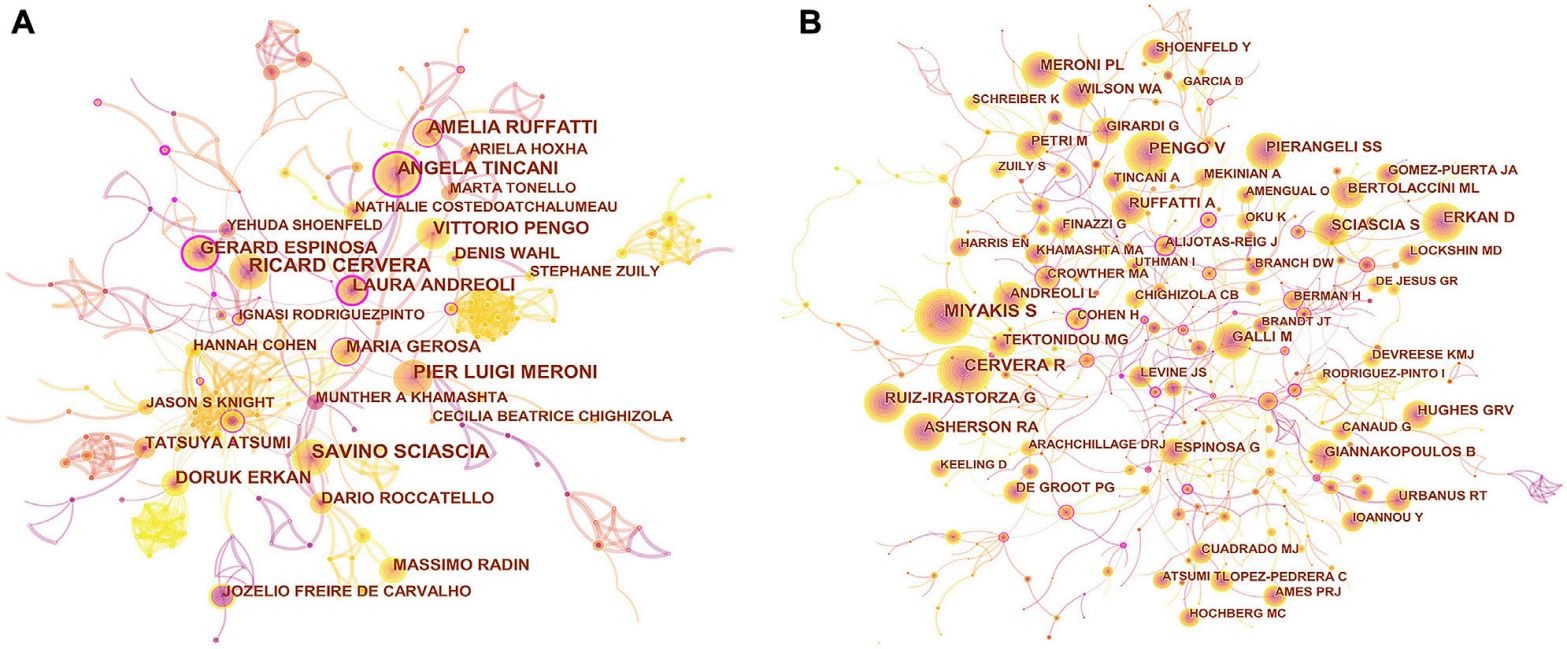
**(A)** CiteSpace visualization map of authors in APS research. **(B)** CiteSpace visualization map of co-cited authors involved in APS. Each circle represents an author, and the size of the circle is proportional to the publication count **(A)** or the number of citations **(B)**. The circles with higher centrality (>0.1) are displayed with purple rings. The lines represent the strength of the co-authorship **(A)** or co-cited relationship **(B)**, with thicker lines representing stronger collaboration **(A)** or relevancy **(B)**. The color of the line represents the time of the first co-authorship, with a more yellow color meaning closer to 2021 and a more red color meaning closer to 2012.

A total of 5,844 authors were responsible for these 1,390 publications. As presented in Table 3, Savino Sciascia is the most prolific author (44, 3.17%), followed by Ricard Cervera (39, 2.81%), Amelia Ruffatti (37, 2.66%), Pier Luigi Meroni (37, 2.66%), and Angela Tincani (36, 2.59%). Figure 3A illustrates that a certain degree of collaboration is observed among the various authors. The authors with the highest centrality were Laura Andreoli (0.34), Gerard Espinosa (0.31), and Angela Tincani (0.27), indicating their important bridging roles in APS research.

**TABLE 3:** Top 10 authors with the most publications involved in APS.

Rank	Author	Count (% of 1,390)	Centrality
1	Savino Sciascia	44 (3.17)	0.04
2	Ricard Cervera	39 (2.81)	0.00
3	Amelia Ruffatti	37 (2.66)	0.11
4	Pier Luigi Meroni	37 (2.66)	0.05
5	Angela Tincani	36 (2.59)	0.27
6	Laura Andreoli	28 (2.01)	0.34
7	Vittorio Pengo	27 (1.94)	0.01
8	Gerard Espinosa	27 (1.94)	0.31
9	Doruk Erkan	25 (1.80)	0.09
10	Maria Gerosa	23 (1.65)	0.17

*Rank: based on the publication count.*

Co-cited authors are two authors cited by another literature at the same time. Table 4 displays that the top 10 co-cited authors are cited > 240 times. Miyakis S owned the most co-citations (962), followed by Cervera R (624), Pengo V (479), Erkan D (364), and Ruiz-Irastorza G (345). Regarding centrality, Sciascia S ranked first with 0.05. Figure 3B shows that Miyakis S is most frequently co-cited with Tektonidou MG, and the connection between the two authors is given in yellow. Notably, none of the top 10 co-cited authors have a purple outer ring (centrality > 0.1).

**TABLE 4:** Top 10 co-cited authors with the most citations involved in APS.

Rank	Co-cited author	Citation	Centrality
1	Miyakis S	962	0.00
2	Cervera R	624	0.01
3	Pengo V	479	0.00
4	Erkan D	364	0.01
5	Ruiz-Irastorza G	345	0.00
6	ASherson RA	327	0.00
7	Sciascia S	299	0.05
8	Meroni PL	286	0.00
9	Pierangeli SS	251	0.00
10	Galli M	242	0.02

*Rank: based on the citation count.*

### VOSviewer for visualization of journals and co-cited journals

**FIGURE 4:**
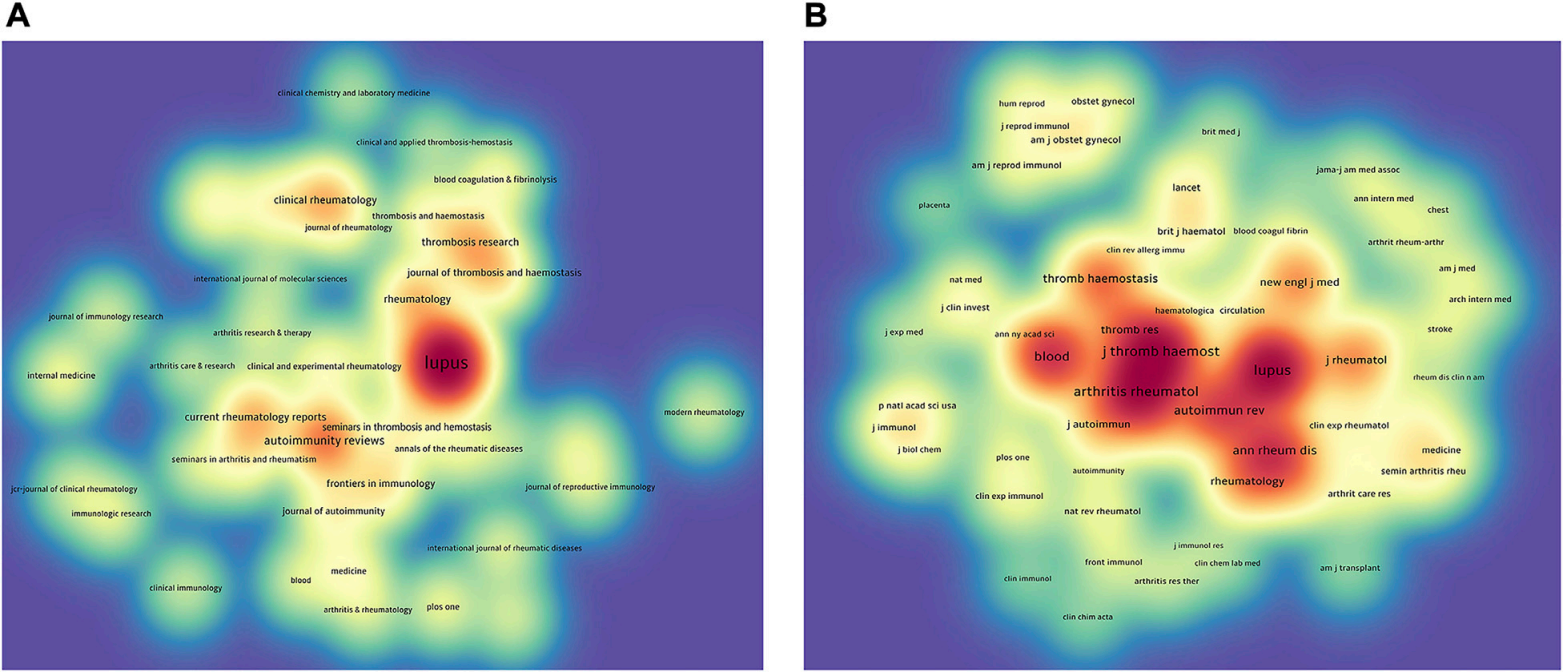
**(A)** VOSviewer density map of journals with publications ≥7 in APS research. The size of the title of the journal is proportional to the publication output. The more publications the journal produces, the closer the bottom background color is to red. **(B)** VOSviewer density map of co-cited journals with citations ≥120 involved in APS. The size of the title of the journal is proportional to the citation count. The more citations a journal receives, the closer the bottom background color is to red.

All documents related to APS research were distributed in 396 journals. Table 5 summarizes that the top 10 most active journals contribute 33.96% of the publications in this area (472). In detail, Lupus offered the highest volume of articles (168, 12.09%), followed by Autoimmunity Reviews (59, 4.24%), Clinical Rheumatology (39, 2.81%), Current Rheumatology Reports (38, 2.73%), and Rheumatology (37, 2.66%). The density map is used to exhibit the journals with publications ≥7 (Figure 4A). Of the top 10 periodicals, seven journals were Q1 in the Journal Citation Reports (JCR) 2021 standards, and Autoimmunity Reviews possessed the highest impact factor (IF; 17.390). In summary, these ten journals laid a firm foundation for future APS research.

**TABLE 5:** Top 10 most productive journals related to APS.

Rank	Journal	Count (% of 1,390)	IF (2021)	JCR (2021)
1	Lupus	168 (12.09)	2.858	Q4
2	Autoimmunity Reviews	59 (4.24)	17.390	Q1
3	Clinical Rheumatology	39 (2.81)	3.650	Q3
4	Current Rheumatology Reports	38 (2.73)	4.686	Q2
5	Rheumatology	37 (2.66)	7.046	Q1
6	Thrombosis Research	35 (2.52)	10.407	Q1
7	Journal of Thrombosis and Haemostasis	29 (2.09)	16.036	Q1
8	Frontiers in Immunology	27 (1.94)	8.786	Q1
9	Journal of Autoimmunity	20 (1.44)	14.511	Q1
10	Seminars in Thrombosis and Hemostasis	20 (1.44)	6.398	Q1/Q2

*Rank: based on the publication count. IF: impact factor. JCR: journal citation reports.*

Table 6 indicates that the most frequently co-cited journal is Lupus (4,012), followed by Arthritis and Rheumatology (3,330), Journal of Thrombosis and Haemostasis (3,247), Blood (2,694), and Autoimmunity Reviews (2,240). Of these top 10 co-cited periodicals, seven journals had citation times surpassing 1,600. A density map is employed to show the co-cited journals with citations ≥120 (Figure 4B). Of these periodicals, the New England Journal of Medicine (New Engl J Med) possessed the highest IF (176.079), followed by Annals of the Rheumatic Diseases (27.973) and Blood (25.476).

**TABLE 6:** Top 10 co-cited journals with the most citations related to APS.

Rank	Co-cited journal	Citation	IF (2021)	JCR (2021)
1	Lupus	4,012	2.858	Q4
2	Arthritis and Rheumatology	3,330	15.483	Q1
3	Journal of Thrombosis and Haemostasis	3,247	16.036	Q1
4	Blood	2,694	25.476	Q1
5	Autoimmunity Reviews	2,240	17.390	Q1
6	Annals of the Rheumatic Diseases	2,234	27.973	Q1
7	Thrombosis and Haemostasis	1,643	6.681	Q1
8	Journal of Rheumatology	1,398	5.346	Q2
9	New England Journal of Medicine	1,384	176.079	Q1
10	Thrombosis Research	1,204	10.407	Q1

*Rank: based on the citation count.*

### Analysis of highly-cited articles

Analyzing highly-cited papers helps to comprehend the basis of disciplinary research. Based on the number of citations, the top 10 most cited publications on APS are exhibited in Table 7. Of them, six belonged to original articles and four belonged to reviews. All these documents were published during 2012–2019, and all of them were cited more than 210 times. The highest number of citations was Giannakopoulos et al. (Giannakopoulos and Krilis, 2013) in the New Engl J Med (394), followed by Andreoli et al. (Andreoli et al., 2017) in Annals of the Rheumatic Diseases (334) and Cervera et al. (Cervera et al., 2015) in Annals of the Rheumatic Diseases (329). These studies were frequently regarded as a knowledge base in APS research.

**TABLE 7:** Top 10 most cited publications on APS.

Rank	Title	First author	Journal	Year	Total citation
1	The pathogenesis of the antiphospholipid syndrome	Giannakopoulos, B	New England Journal of Medicine	2013	394
2	EULAR recommendations for women’s health and the management of family planning, assisted reproduction, pregnancy and menopause in patients with systemic lupus erythematosus and/or antiphospholipid syndrome	Andreoli, L	Annals of the Rheumatic Diseases	2017	334
3	Morbidity and mortality in the antiphospholipid syndrome during a 10-year period: a multicentre prospective study of 1,000 patients	Cervera, R	Annals of the Rheumatic Diseases	2015	329
4	Rivaroxaban vs. warfarin in high-risk patients with antiphospholipid syndrome	Pengo, V	Blood	2018	311
5	EULAR recommendations for the management of antiphospholipid syndrome in adults	Tektonidou, MG	Annals of the Rheumatic Diseases	2019	297
6	Guidelines on the investigation and management of antiphospholipid syndrome	Keeling, D	British Journal of Haematology	2012	288
7	Diagnosis and management of the antiphospholipid syndrome	Garcia, D	New England Journal of Medicine	2018	270
8	The hyperferritinemic syndrome: macrophage activation syndrome, Still’s disease, septic shock and catastrophic antiphospholipid syndrome	Rosario, C	BMC Medicine	2013	266
9	Rivaroxaban *versus* warfarin to treat patients with thrombotic antiphospholipid syndrome, with or without systemic lupus erythematosus (RAPS): a randomised, controlled, open-label, phase 2/3, non-inferiority trial	Cohen, H	Lancet Haematology	2016	225
10	Antiphospholipid syndrome	Schreiber, K	Nature Reviews Disease Primers	2018	215

*Rank: based on the citation count.*

### CiteSpace for visualization of co-cited references and references burst

**FIGURE 5:**
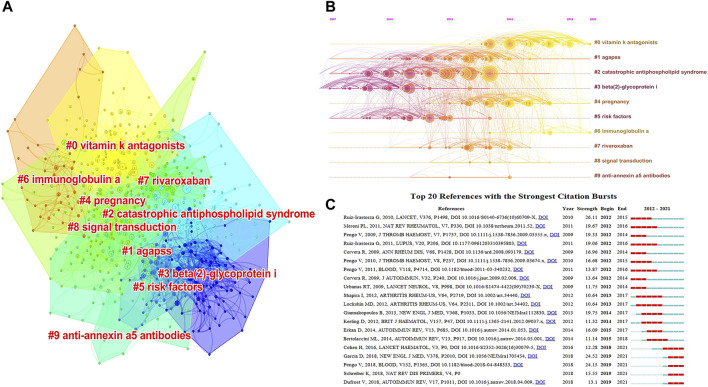
**(A)** Ten clustering maps of reference co-citation analysis based on CiteSpace. Each cluster is represented by a different color, and the smaller the number label, the larger the cluster profile and the more co-cited references (dots) it contains. **(B)** Timeline view map of reference co-citation analysis generated by CiteSpace. The elements on the horizontal axis represent co-cited references; the position of the element on the horizontal axis denotes the time of the first occurrence; and the line connecting the elements denotes the co-cited relationship. The size of the element is proportional to the citation count of the reference. A more yellow color indicates closer to the year 2021, and a more red color indicates closer to the year 2012. **(C)** Top 20 references with the strongest citation bursts by CiteSpace. Each rectangle represents a year. Green rectangles indicate the time interval, and red rectangles indicate the duration of the burst from the beginning to the end.

Reference co-citation analysis is a significant tool for exploring the evolution and discovering the developmental frontiers in a given domain. CiteSpace is utilized for clustering analysis of co-cited references, and the whole network map is classified into 10 distinct clusters based on the log-likelihood ratio (LLR) algorithm (Figure 5A). In cluster analysis, the mean silhouette (S) value is an index of cluster homogeneity, and S >0.7 denotes the conviction of the clustering results. The modularity (Q) value is an index of the degree of grouping of nodes, and Q >0.3 denotes the significance of the clustering structure (Chen et al., 2012). The Q value was 0.5902 and the S value was 0.8428 in this work, indicating the reliability of the results. As described in Figure 5A, each cluster is represented by a different color, and the smaller the number label, the larger the cluster profile, meaning that the cluster contains more co-cited references (dots). Table 8 illustrates that “vitamin K antagonists (VKA)” is the largest cluster (#0), followed by “agapss” (#1), “CAPS” (#2), and “β2GPI” (#3). The timeline view in Figure 5B presents the evolution process of each cluster. The elements on the horizontal axis represent co-cited references; the position of the element on the horizontal axis denotes the time of the first occurrence; and the line connecting the elements denotes the co-cited relationship. The size of the element is proportional to the citation count of the reference. A more yellow color indicates closer to the year 2021, and a more red color indicates closer to the year 2012. The research hotspots have shifted from “β2GPI” (#3), “risk factors” (#5), and “CAPS” (#2) to “pregnancy” (#4), “VKA” (#0), and “immunoglobulin A (IgA)” (#6).

**TABLE 8:** The clusters information of co-cited references on APS.

Cluster ID	Size	Silhouette	Label (LLR)	Mean year
^#^0	116	0.865	Vitamin K antagonists	2017
^#^1	106	0.797	Agapss	2014
^#^2	100	0.831	Catastrophic antiphospholipid syndrome	2011
^#^3	87	0.842	Beta (2)-glycoprotein I	2009
^#^4	86	0.791	Pregnancy	2015
^#^5	55	0.835	Risk factors	2010
^#^6	39	0.903	Immunoglobulin A	2018
^#^7	36	0.912	Rivaroxaban	2014
^#^8	19	0.944	Signal transduction	2015
^#^9	6	0.994	Anti-annexin A5 antibodies	2013

*Rank: based on the size. “Size” denotes the amount of co-cited references that a cluster contains. LLR: log-likelihood ratio.*

Burst detection is a useful approach for capturing rapid increases in the popularity of references or keywords, which denotes that the topic has received great attention over a set period. Figure 5C lists the top 20 references with the strongest citation bursts. The red segments in this picture signify the duration of the reference outbreak, and the green lines denote the time period. Of these co-cited references, Ruiz-Irastorza et al. (Ruiz-Irastorza et al., 2010) in Lancet had the strongest strength of burst (26.11), followed by Garcia et al. (Garcia and Erkan, 2018) in the New Engl J Med (24.52) and Pengo et al. (Pengo et al., 2018) in Blood (24.15). Notably, of these top 20 references, articles from Pengo V occupied one-fifth of the seats (4), demonstrating the profound influence of Pengo V in the APS field. While the outbreak of most references has ended, there are still five references in the citation burst, implying that these areas are research directions to focus on in the future.

### VOSviewer for visualization of keywords co-occurrence and evolution and CiteSpace for visualization of keywords burst

**FIGURE 6:**
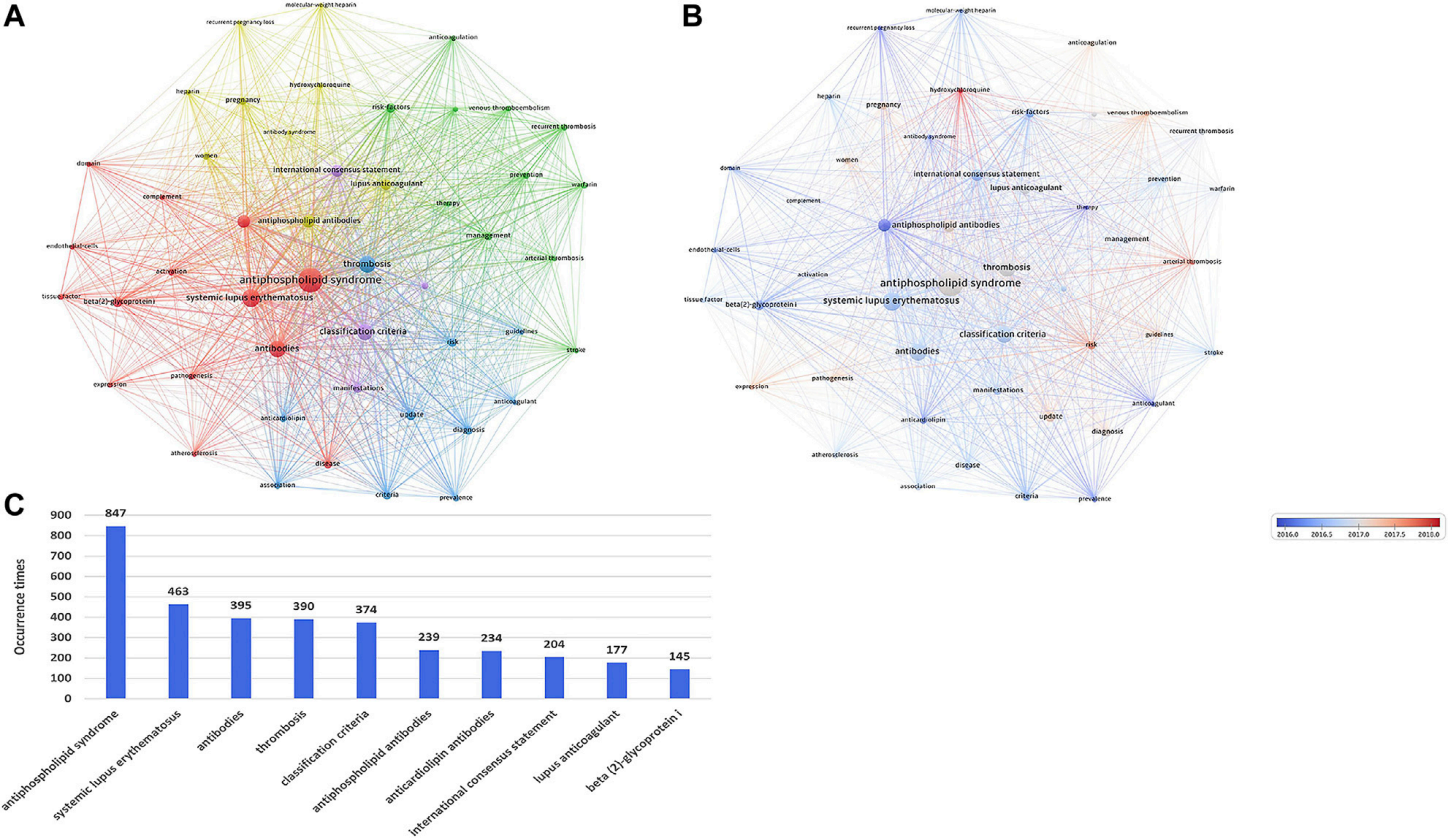
**(A)** Network clustering map of keywords’ co-occurrence analysis based on VOSviewer. The minimum threshold for the number of keyword frequencies is 40. Keywords were divided into 5 clusters: cluster 1 (red), cluster 2 (green), cluster 3 (blue), cluster 4 (yellow), and cluster 5 (purple). Each keyword is represented as a node, and the node size is proportional to the frequency. The line between nodes denotes a co-occurrence relationship. The distance between nodes denotes the degree of relevancy, and the closer the distance, the higher the degree of relevancy. **(B)** Overlay map of keywords’ co-occurrence analysis based on VOSviewer. The node color denotes the corresponding average occurrence time according to the color gradient in the lower right corner. Nodes displayed in blue represent keywords that appear relatively early, while nodes displayed in red represent keywords that appear later. **(C)** Top 20 keywords with the highest occurrence times.

The co-occurrence analysis of keywords reveals the dominating motifs within a specific field. In the clustering analysis, after merging keywords with the same content, 48 items were identified and classified into 5 clusters (minimum amount of frequencies of a keyword ≥40). As exhibited in Figure 6A, a keyword is represented as a node, and the size of the node reflects the frequency of the keyword.

The line between nodes denotes a co-occurrence relationship. The distance between nodes denotes the degree of relevancy, and the closer the distance, the higher the degree of relevancy. The five clusters, which were centered on APS, risk factors, thrombosis, aPL, and classification criteria, were each marked with a different color. Nodes with comparable characteristics were split into a color-marked cluster and displayed in red (cluster 1, studies on pathogenesis), green (cluster 2, studies on risk factors and prevention), blue (cluster 3, studies on diagnosis), yellow (cluster 4, studies on treatment), and purple (cluster 5, studies on classification).

Figure 6B illustrates the dynamic evolution of keywords over time. Keywords that presented comparatively earlier are marked in blue, while keywords that emerged relatively later are marked in red. Keywords such as “therapy,” “aCL antibodies,” “recurrent pregnancy loss,” “anticoagulant,” and “antibody syndrome” were the major topics in the early stage. Keywords such as “venous thromboembolism (VTE),” “expression,” “risk,” “arterial thrombosis (AT),” and “hydroxychloroquine (HCQ)” evolved more recently, implying that these themes are attracting much attention at present.

**FIGURE 7:**
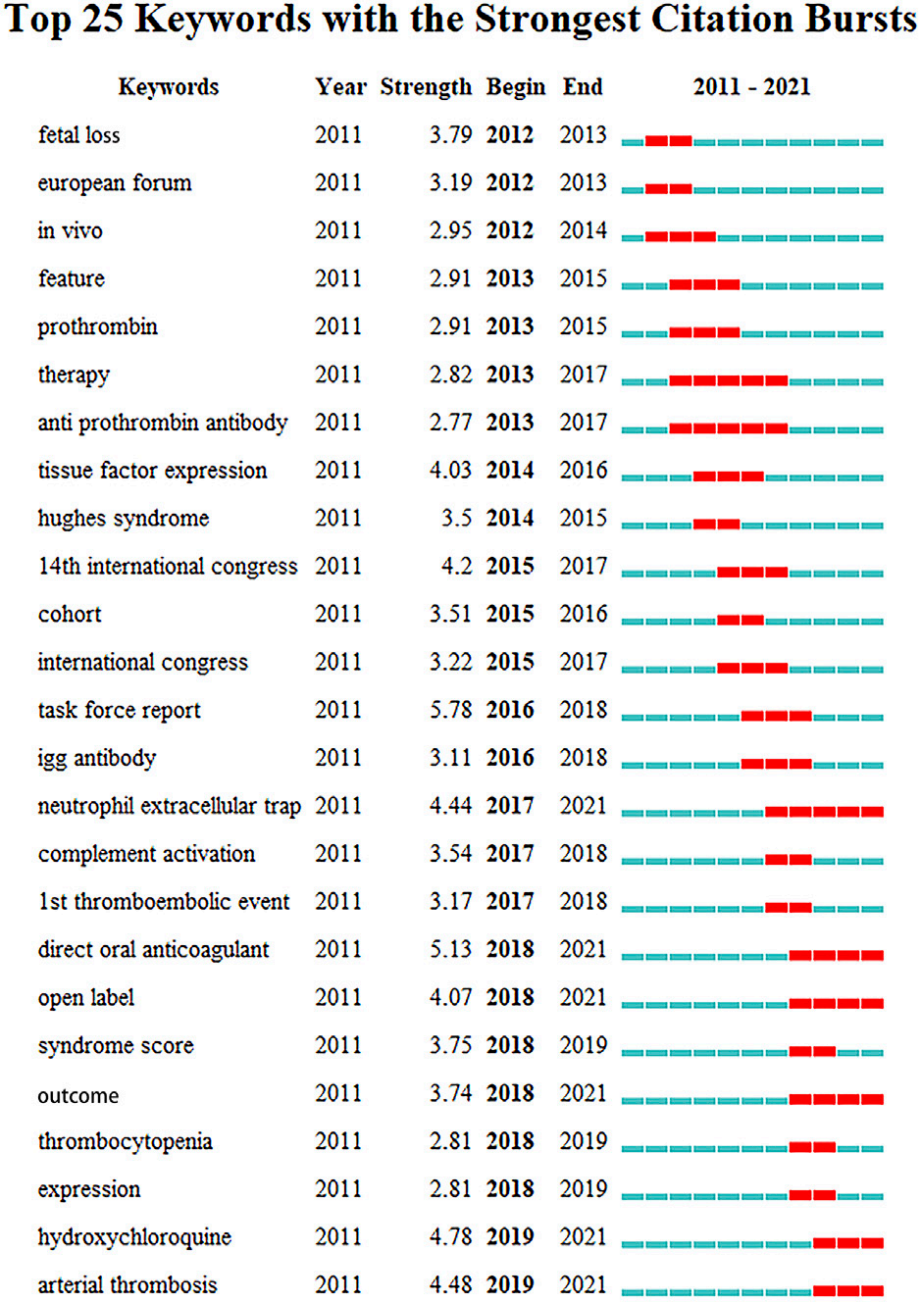
Top 25 keywords with the strongest citation bursts by CiteSpace. Each rectangle represents a year. Green rectangles indicate the time interval, and red rectangles indicate the duration of the burst from the beginning to the end.

As shown in Figure 6C, the top 10 keywords appear >140 times. The most commonly occurring keyword was APS (847), followed by SLE (463), antibodies (395), thrombosis (390), and classification criteria (374). Figure 7 presents the top 25 burst keywords. From 2017 to the present, the terms “neutrophil extracellular trap (NET),” “direct oral anticoagulant (DOAC),” “open label,” “outcome,” “HCQ,” and “AT” are still in the outbreak period.

## Discussion

### General information

Through bibliometrics, this study performs a systematic and visual analysis of the APS field and detects the research status and hotspots in the domain. The annual literature volume and trend can reveal the pace of development in a given field. According to qualitative and quantitative analysis using CiteSpace and VOSviewer software, scientific publications related to APS have been increasing continuously over the last 10 years. While the number of documents fluctuated and declined at certain time points, the overall trend gradually increased and reached its peak in 2021, with 175 papers, suggesting that the field of APS has attracted increasing attention from experts and scholars. In the work of Deng et al. and Liu et al., similar increasing trends were described (Deng et al., 2022b; Liu et al., 2022).

As shown in Table 1, Italy is the most productive country (273, 19.64%), followed by the United States (258, 18.56%) and the United Kingdom (155, 11.15%), which together account for 49.35% of the total. However, after adjusting for population size, Israel ranked on top, with 7.27 articles per million people. Of note, apart from Brazil and China, the rest of the top 10 countries all belong to developed countries. The main causes of differences in literature output between countries may be attributed to disparities in socioeconomic position, general research ability, and population size (Fan et al., 2017). In addition, only one of the top 10 countries had a centrality greater than 0.1, implying a lack of strong influence from these countries in the domain of APS. Regarding institutional contributions (Table 2), the University of Padua (Italy) had the greatest number of publications (69, 4.96%), whereas the University of Milan and the University College London ranked first in centrality value with 0.27, suggesting the high impact of these institutions in the field of APS. Among the top 10 institutions, eight were from developed countries, and there were four organizations from Italy, which helped reveal why Italy has been a leader in this field for the past decade. As shown in Figure 2C and Figure 2D, the close cooperation among countries and institutions is mainly focused on developed countries. To better foster the prosperity of this field, geographical barriers should be broken down, and exchanges and collaboration between developed and developing countries in this field should be further strengthened.

For authors (Table 3) and co-cited authors (Table 4), Savino Sciascia (44, 3.17%) contributes the most documents, while Miyakis S owns the most co-citations (962). Five authors had a crucial bridging role among the top 10 authors (centrality >0.1). Notably, although Miyakis S had not published many papers, he topped the list of the top 10 co-cited authors. This phenomenon may be attributed to the high-impact article he has contributed (Miyakis et al., 2006). Professor Savino Sciascia is from San Giovanni Bosco Hospital in Italy. In 2013, Savino Sciascia and his colleagues developed and validated a risk score [global APS score (GAPSS)] in patients with SLE (Sciascia et al., 2013). The use of GAPSS significantly improved the prediction of the risk of thrombosis or pregnancy loss in SLE and transformed the concept of aPL as diagnostic antibodies to aPL as a risk factor for clinical events. In 2015, Savino Sciascia and his colleagues evaluated the clinical relevance of GAPSS in primary APS patients (Sciascia et al., 2015). They found higher GAPSS values in patients with only thrombosis compared to those with only pregnancy loss; individuals with recurrent thrombosis had higher GAPSS values than those without recurrence. They proposed that GAPSS values ≥11 were closely related to a higher risk of thrombotic recurrence. The study showed that the GAPSS was a useful method that significantly enhanced risk stratification regarding thrombosis in primary APS.

The analysis of journals indicated that Lupus is the most active periodical (168, 12.09%), followed by Autoimmunity Reviews (59, 4.24%) and Clinical Rheumatology (39, 2.81%) (Table 5). Scholars interested in APS research can pay more attention to these magazines because of their large volume of publications on APS. Regarding the sources of these top 10 magazines, nine are from countries in Western Europe and North America. None of the journals from Asian countries appeared on the list. Thus, Asian countries may enhance the construction of journals in the field of APS. For co-cited journals (Table 6), Lupus was the journal with the highest citations (4,012), followed by Arthritis and Rheumatology (3,330), Journal of Thrombosis and Haemostasis (3,247), Blood (2,694), and Autoimmunity Reviews (2,240). Of note, Lupus came out on top in both the most published and most co-cited journals, demonstrating its dominance in the area of APS. Of these top 10 co-cited journals, eight periodicals were located in the Q1 JCR region, implying that high-impact magazines had an interest in APS-related research. In addition, a 40% agreement rate is achieved between the top 10 periodicals and co-cited periodicals, implying a certain degree of focus on the simultaneous development of quantity and quality in these journals.

The impact of a publication can be reflected by the number of citations to some extent. Table 7 displays the top 10 most cited documents on APS. Specifically, Giannakopoulos et al. published “The pathogenesis of the antiphospholipid syndrome” in the New Engl J Med in 2013 (Giannakopoulos and Krilis, 2013), which was the most cited study (394). This essay provided a detailed overview of the thrombotic mechanisms of APS, including post-translational redox modifications of β2GPI, conformations of β2GPI, the “two hit” model, endothelial nitric oxide synthase, endothelial cells and monocytes, tissue factor, factor XI, platelets, annexin A5 anticoagulant shield and HCQ, complement and neutrophils, and disturbance of innate immunity, which were involved in the pathophysiology of APS. In summary, the top 10 publications with the highest citations were focused on the following topics: pathogenesis, the management of pregnancy, epidemiology, diagnosis and treatment.

### The analysis of hotspots and frontiers

Reference co-citation analysis is a useful method to evaluate progress and identify hotspots within a specific field. As shown in Table 8, before 2014, the topics appearing more often are “β2GPI” (^#^3), “risk factors” (^#^5), “CAPS” (^#^2), and “anti-annexin A5 antibodies” (^#^9). After 2017, the more common subjects are “VKA” (^#^0) and “IgA” (^#^6), implying that the issues of the above clusters are the current research focuses in the domain. As the third most frequent cause of mortality worldwide (Bitsadze et al., 2022), VTE is considered to be the most common clinical feature of APS. VKA, primarily warfarin, is recognized as the gold standard for secondary prevention of thrombotic APS (Fujieda and Amengual, 2020). In individuals with APS and a first onset of VTE, a VKA with an international normalized ratio (INR) aim of two to three is recommended (Tektonidou et al., 2019). Proof from previous studies indicated that higher anticoagulation strength (INR 3.0–4.5) provided no additional benefit (Crowther et al., 2003; Finazzi et al., 2005). Due to the risk of bleeding with long-term VKA use, regular INR monitoring is essential. Prothrombin time-INR (PT-INR) is the standard way for monitoring the anticoagulant strength of VKA. However, in LA-positive APS patients, thromboplastin can be affected, resulting in PT-INR being raised (Cohen et al., 2021). Thus, more effective anticoagulation monitoring tools merit additional consideration. IgA isotypes of aPL are not presently included in standard diagnostic procedures for APS, and their value as a diagnostic marker is still up for debate (Sciascia et al., 2017). Several studies have demonstrated a link between the IgA isotypes and thrombosis (Pericleous et al., 2016; Tebo et al., 2016). Recently, Reshetnyak et al. reported that IgA aCL and IgA aβ2GPI had a high specificity (95% and 93%) but a low sensitivity (54% and 44%) in detecting APS (Reshetnyak et al., 2022). The diagnostic value of IgA aPL is worthy of further study in the future. The timeline view of co-cited references visually displays the evolutionary trajectory of each cluster (Figure 5B).

References with citation bursts mean the sudden rise of citations of specific publications in a given stage, which can serve as a practical technique to find emerging themes attracting great interest during some period of time. The top 20 references with the strongest citation bursts are presented in Figure 5C. It is worth noting that while the bursts in most references were finished, five documents (20%) in the top 20 remain in a state of burst, focusing on the diagnosis and treatment of APS, suggesting that these research topics are the most up-to-date ones at the moment. Of these five papers, the essay with the highest burst strength (24.52) was published by Garcia et al. (Garcia and Erkan, 2018) in the New Engl J Med in 2018. This paper expounded on evidence-based recommendations for the recognition and diagnosis of APS, as well as therapy recommendations for patients with persistently positive aPL. Pengo et al. (Pengo et al., 2018) offered the second-ranked essay (24.15), which compared the efficacy and safety of rivaroxaban *versus* warfarin in high-risk triple-positive (presence of LA, aβ2GP1, and aCL) APS patients. The results showed 11 (19%) events in the rivaroxaban arm and 2 (3%) in the warfarin arm. Finally, the trial was terminated early due to an excess of events in patients in the rivaroxaban group. Schreiber et al. (Schreiber et al., 2018) offered the third-ranked essay (15.55), which described the pathogenesis, diagnosis, and treatment of APS in detail and provided an outlook on future research subjects. Dufrost et al. (Dufrost et al., 2018) offered the fourth-ranked essay (13.1), which evaluated the prevalence of recurrent thrombosis in patients with APS receiving DOACs treatment and detected risk factors related to recurrent thrombosis. The results indicated a higher thrombotic risk in a portion of individuals with APS treated with DOACs. Cohen et al. (Cohen et al., 2016) offered the fifth-ranked essay (12.28), which compared the efficacy and safety of rivaroxaban and warfarin in patients with thrombotic APS for the first time. The results showed that compared to the use of warfarin, the use of rivaroxaban resulted in a 100% increase in endogenous thrombin potential and a 40% reduction in peak thrombin generation, with a similar incidence of adverse effects.

In addition to references, keywords also serve as a representation of the main motifs and core contents in a given field. Based on the co-occurrence analysis of high-frequency keywords (Figure 6A), five current research directions for APS were recognized, which are as follows: “the pathogenesis of APS,” “the prevention and risk factors for APS,” “the research on the diagnosis of APS,” “the treatment of APS” and “the classification for APS.” Moreover, we conduct the evolution process of high-frequency keywords to better understand the mutative course of the APS research themes (Figure 6B). Keywords are marked with various colors depending on the average occurrence time of the items, that is, red keywords arise on average later than blue keywords. In the early period, the majority of blue keywords appeared in cluster 3 (blue, focusing on diagnosis) and cluster 5 (purple, focusing on classification). In recent years, most of the red keywords appear in cluster 1 (red, focusing on pathogenesis), cluster 2 (green, focusing on risk factors and prevention) and cluster 4 (yellow, focusing on treatment), suggesting that after 2018, more studies will be concentrated on the pathogenesis, risk factors and prevention and treatment of APS.

Furthermore, we identified the top 25 burst keywords between 2012 and 2021. Of these, we primarily focus on those topics that continue to explode into 2021, which indicates that these subjects are potential research frontiers for the future. As shown in Figure 7, these keywords are mainly associated with clinical studies on the treatment and mechanism research of APS, including NET, DOAC, open label, outcome, HCQ, and AT. Neutrophils contribute to thrombosis by releasing substances into the extracellular space, primarily DNA and histones, defined as NETs (Meng et al., 2017). A cross-sectional study found that NETs contribute to activated protein C resistance, which plays a role in the hypercoagulable condition of APS individuals (Foret et al., 2022). Mazetto et al. (Mazetto et al., 2022) recently reported that patients with thrombotic APS have elevated levels of gene expression associated with neutrophil activity and the release of NETs, which may be involved in thrombus formation.

In terms of treatment, as mentioned earlier, warfarin is considered the gold standard for secondary prevention of thrombotic APS (Fujieda and Amengual, 2020). However, the presence of APS patients on warfarin requiring frequent blood draws to monitor INR and warfarin intolerance has led to considerable interest in the use of DOACs instead of VKA (Ghembaza and Saadoun, 2020). The major benefits of DOACs *versus* VKA are their standardized dose regimen, faster and more predictable anticoagulant reaction, absence of frequent laboratory monitoring, and less major bleeding (Wiggins et al., 2020). Several recent randomized controlled and open-label studies have compared the efficacy of DOACs *versus* VKA in patients with APS (Cohen et al., 2016; Pengo et al., 2018; Ordi-Ros et al., 2019), and their outcomes appear to be contradictory, but it is worth noting that these trials were conducted in diverse APS populations. The results of Pengo et al.’s study (all of 120 patients were triple aPL positive) indicated that 12% of those using rivaroxaban experienced thromboembolic events, compared to 0% of those taking warfarin (Pengo et al., 2018). The outcomes of Cohen et al.’s study (28% of 116 patients were triple aPL positive) showed that neither the VKA nor rivaroxaban groups had experienced recurrent thrombosis after 210 days of follow-up (Cohen et al., 2016). The results of Ordi-Ros et al.’s study (61% of 190 patients were triple aPL positive) suggested that the VKA and rivaroxaban groups had no statistical difference in the rate of recurrent thrombosis, although higher AT occurred in the rivaroxaban group (Ordi-Ros et al., 2019). A more recent meta analysis of seven randomised controlled studies demonstrated that VKA is a more effective treatment option for individuals with APS than DOACs, especially rivaroxaban, given that DOACs use is linked to a 69% higher risk of thromboembolic events (Koval et al., 2021). To demonstrate the effectiveness of DOACs in different subgroups of APS patients and different types of DOACs for the treatment of APS, further high-quality studies are warranted.

Moreover, additional treatment with HCQ, an antimalarial agent, has been frequently mentioned in the literature in recent years. HCQ has already been demonstrated to reduce the risk of thrombosis in individuals with SLE (Petri, 2011). Kravvariti et al. (Kravvariti et al., 2020) reported the results of a randomized controlled study, which suggested that HCQ could also reduce the incidence of thrombosis and lower APL titers in APS patients. A similar result was reported in a retrospective study by Nuri et al. (Nuri et al., 2017), which found that HCQ could also decrease APL levels and significantly diminish the recurrence rate of AT in individuals with APS. Several studies have attributed the thromboprophylaxis effects of HCQ to its anti-inflammatory, immunoregulatory, metabolic and antithrombotic properties (Ben-Zvi et al., 2012; Andrade and Tektonidou, 2016).

Stroke and transient ischaemic attack (TIA) are the two symptoms of APS that occur most frequently in the arterial circulation. It was reported that the prevalence of stroke and TIA in individuals with APS was 13.1% and 7.0%, respectively (Cervera et al., 2002). An analysis of five cohort studies suggested that low-dose aspirin reduced the risk of first AT in patients with aPL (Arnaud et al., 2015). A greater probability of recurrence exists in APS patients with AT compared to those with VTE. However, the optimal anticoagulant target after AT is still up for debate. For individuals with AT, the Galveston guidelines suggested either high-intensity warfarin at an INR >3.0 or combined treatment with low-dose aspirin plus warfarin (INR 2.0–3.0) (Ruiz-Irastorza et al., 2011). Adequate and robust antithrombotic treatment strategies after AT is an urgent issue that needs to be addressed.

### Limitations

The study had several limitations. First, although limiting the search to titles can improve the accuracy and relevance of our search results, it is inevitable that articles related to the topic may be missed, and using a combination of titles and logical operators may further improve the accuracy of the search results (Cheng et al., 2022a; 2022b). Second, the bibliometric information included only data from the WoSCC database, leaving out data from other major databases, which might result in the exclusion of a few relevant studies. Third, we looked only at documents published in English and excluded those published in other languages, which indicated that non-English speaking countries’ contributions may be neglected to some extent. Last, the data extraction took place on 24 May 2022, which might slightly influence the analysis results by the time.

## Conclusions

This is the first bibliometric analysis of the quantity and quality of APS-related publications, which suggests that global research on APS has increased continuously over the past 10 years. Globally, Italy and the United States are the leading countries in the field. The University of Padua, the University of Milan, and the University of Sao Paulo are the top three most prolific institutions. Lupus ranks first in both the most published and most co-cited journals. Savino Sciascia and Spiros Miyakis are the most productive and most co-cited authors, respectively. Notably, “VKA” and “IgA” are current research foci in the domain of APS. The clinical studies on the treatment and mechanism research of APS are recognized as promising research frontiers. In conclusion, this bibliometric study presents the current research status and frontiers in the APS field, which can explore potential collaboration opportunities and provide a reference for subsequent investment decisions and research directions.

## Data Availability

The original contributions presented in the study are included in the article/supplementary material, further inquiries can be directed to the corresponding author.

## Abbreviations

aCL, anticardiolipin; AT, arterial thrombosis; APS, antiphospholipid syndrome; aβ2GPI, anti-beta two glycoprotein I; aPL, antiphospholipid antibodies; CAPS, catastrophic antiphospholipid syndrome; COVID-19, coronavirus disease 2019; DOAC, direct oral anticoagulant; GAPSS, global antiphospholipid syndrome score; HCQ, hydroxychloroquine; IF, impact factor; IgA, immunoglobulin A; INR, international normalized ratio; JCR, Journal Citation Reports; LA, lupus anticoagulant; LLR, log-likelihood ratio; NET, neutrophil extracellular trap; PT, prothrombin time; SLE, systemic lupus erythematosus; Q, modularity; S, silhouette; SCI-Expanded, Science Citation Index Expanded; TIA, transient ischaemic attack; TI, title; New Engl J Med, New England Journal of Medicine; VKA, vitamin K antagonists; VTE, venous thromboembolism; WoS, Web of Science; WoSCC, Web of Science Core Collection.

## References

[B1] Aksoy U. Kucuk M. Versiani M. A. Orhan K. (2021). Publication trends in micro-CT endodontic research: A bibliometric analysis over a 25-year period. Int. Endod. J. 54 (3), 343–353. 10.1111/iej.13433 33075147

[B2] Andrade D. Tektonidou M. (2016). Emerging therapies in antiphospholipid syndrome. Curr. Rheumatol. Rep. 18 (4), 22. 10.1007/s11926-016-0566-z 26995745

[B3] Andreoli L. Bertsias G. K. Agmon-Levin N. Brown S. Cervera R. Costedoat-Chalumeau N. (2017). EULAR recommendations for women’s health and the management of family planning, assisted reproduction, pregnancy and menopause in patients with systemic lupus erythematosus and/or antiphospholipid syndrome. Ann. Rheum. Dis. 76 (3), 476–485. 10.1136/annrheumdis-2016-209770 27457513 PMC5446003

[B4] Arnaud L. Mathian A. Devilliers H. Ruffatti A. Tektonidou M. Forastiero R. (2015). Patient-level analysis of five international cohorts further confirms the efficacy of aspirin for the primary prevention of thrombosis in patients with antiphospholipid antibodies. Autoimmun. Rev. 14 (3), 192–200. 10.1016/j.autrev.2014.10.019 25461472

[B5] Ben-Zvi I. Kivity S. Langevitz P. Shoenfeld Y. (2012). Hydroxychloroquine: From malaria to autoimmunity. Clin. Rev. Allergy Immunol. 42 (2), 145–153. 10.1007/s12016-010-8243-x 21221847 PMC7091063

[B6] Bitsadze V. Khizroeva J. Alexander M. Elalamy I. (2022). Venous thrombosis risk factors in pregnant women. J. Perinat. Med. 50 (5), 505–518. 10.1515/jpm-2022-0008 35044114

[B7] Borghi M. O. Beltagy A. Garrafa E. Curreli D. Cecchini G. Bodio C. (2020). Anti-phospholipid antibodies in COVID-19 are different from those detectable in the anti-phospholipid syndrome. Front. Immunol. 11, 584241. 10.3389/fimmu.2020.584241 33178218 PMC7593765

[B8] Cervera R. (2017). Antiphospholipid syndrome. Thromb. Res. 151 (1), S43–S47. 10.1016/S0049-3848(17)30066-X 28262233

[B9] Cervera R. Piette J. C. Font J. Khamashta M. A. Shoenfeld Y. Camps M. T. (2002). Antiphospholipid syndrome: Clinical and immunologic manifestations and patterns of disease expression in a cohort of 1, 000 patients. Arthritis Rheum. 46 (4), 1019–1027. 10.1002/art.10187 11953980

[B10] Cervera R. Rodriguez-Pinto I. Espinosa G. (2018). The diagnosis and clinical management of the catastrophic antiphospholipid syndrome: A comprehensive review. J. Autoimmun. 92, 1–11. 10.1016/j.jaut.2018.05.007 29779928

[B11] Cervera R. Serrano R. Pons-Estel G. J. Ceberio-Hualde L. Shoenfeld Y. de Ramon E. (2015). Morbidity and mortality in the antiphospholipid syndrome during a 10-year period: A multicentre prospective study of 1000 patients. Ann. Rheum. Dis. 74 (6), 1011–1018. 10.1136/annrheumdis-2013-204838 24464962

[B12] Chen C. Hu Z. Liu S. Tseng H. (2012). Emerging trends in regenerative medicine: A scientometric analysis in CiteSpace. Expert Opin. Biol. Ther. 12 (5), 593–608. 10.1517/14712598.2012.674507 22443895

[B13] Cheng K. Guo Q. Shen Z. Yang W. Wang Y. Sun Z. (2022a). Bibliometric analysis of global research on cancer photodynamic therapy: Focus on nano-related research. Front. Pharmacol. 13, 927219. 10.3389/fphar.2022.927219 35784740 PMC9243586

[B14] Cheng K. Guo Q. Yang W. Wang Y. Sun Z. Wu H. (2022b). Mapping knowledge landscapes and emerging trends of the links between bone metabolism and diabetes mellitus: A bibliometric analysis from 2000 to 2021. Front. Public Health 10, 918483. 10.3389/fpubh.2022.918483 35719662 PMC9204186

[B15] Cohen H. Efthymiou M. Devreese K. M. J. (2021). Monitoring of anticoagulation in thrombotic antiphospholipid syndrome. J. Thromb. Haemost. 19 (4), 892–908. 10.1111/jth.15217 33325604

[B16] Cohen H. Hunt B. J. Efthymiou M. Arachchillage D. R. J. Mackie I. J. Clawson S. (2016). Rivaroxaban versus warfarin to treat patients with thrombotic antiphospholipid syndrome, with or without systemic lupus erythematosus (raps): A randomised, controlled, open-label, phase 2/3, non-inferiority trial. Lancet. Haematol. 3 (9), e426–e436. 10.1016/S2352-3026(16)30079-5 27570089 PMC5010562

[B17] Cohen H. Isenberg D. A. (2021). How I treat anticoagulant-refractory thrombotic antiphospholipid syndrome. Blood 137 (3), 299–309. 10.1182/blood.2020004942 32898856

[B18] Crowther M. A. Ginsberg J. S. Julian J. Denburg J. Hirsh J. Douketis J. (2003). A comparison of two intensities of warfarin for the prevention of recurrent thrombosis in patients with the antiphospholipid antibody syndrome. N. Engl. J. Med. 349 (12), 1133–1138. 10.1056/NEJMoa035241 13679527

[B19] Deng P. Shi H. Pan X. Liang H. Wang S. Wu J. (2022a). Worldwide research trends on diabetic foot ulcers (2004-2020): Suggestions for researchers. J. Diabetes Res. 2022, 7991031. 10.1155/2022/7991031 35127951 PMC8813288

[B20] Deng P. Wang S. Sun X. Qi Y. Ma Z. Pan X. (2022b). Global trends in research of gouty arthritis over past decade: A bibliometric analysis. Front. Immunol. 13, 910400. 10.3389/fimmu.2022.910400 35757713 PMC9229989

[B21] Duarte-Garcia A. Pham M. M. Crowson C. S. Amin S. Moder K. G. Pruthi R. K. (2019). The epidemiology of antiphospholipid syndrome: A population-based study. Arthritis Rheumatol. 71 (9), 1545–1552. 10.1002/art.40901 30957430 PMC6717037

[B22] Dufrost V. Risse J. Reshetnyak T. Satybaldyeva M. Du Y. Yan X. X. (2018). Increased risk of thrombosis in antiphospholipid syndrome patients treated with direct oral anticoagulants. Results from an international patient-level data meta-analysis. Autoimmun. Rev. 17 (10), 1011–1021. 10.1016/j.autrev.2018.04.009 30103045

[B23] Fan G. Han R. Zhang H. He S. Chen Z. (2017). Worldwide research productivity in the field of minimally invasive spine surgery: A 20-year survey of publication activities. Spine 42 (22), 1717–1722. 10.1097/BRS.0000000000001393 26679875

[B24] Finazzi G. Marchioli R. Brancaccio V. Schinco P. Wisloff F. Musial J. (2005). A randomized clinical trial of high-intensity warfarin vs. Conventional antithrombotic therapy for the prevention of recurrent thrombosis in patients with the antiphospholipid syndrome (WAPS). J. Thromb. Haemost. 3 (5), 848–853. 10.1111/j.1538-7836.2005.01340.x 15869575

[B25] Foret T. Dufrost V. Salomon du Mont L. Costa P. Lakomy C. Lagrange J. (2022). A new pro-thrombotic mechanism of neutrophil extracellular traps in antiphospholipid syndrome: Impact on activated protein C resistance. Rheumatology 61 (7), 2993–2998. 10.1093/rheumatology/keab853 34791113

[B26] Foret T. Dufrost V. Salomon Du Mont L. Costa P. Lefevre B. Lacolley P. (2021). Systematic review of antiphospholipid antibodies in COVID-19 patients: Culprits or bystanders? Curr. Rheumatol. Rep. 23 (8), 65. 10.1007/s11926-021-01029-3 34218350 PMC8254447

[B27] Fujieda Y. Amengual O. (2020). New insights into the pathogenic mechanisms and treatment of arterial thrombosis in antiphospholipid syndrome. Eur. J. Rheumatol. 8 (2), 93–99. 10.5152/eurjrheum.2020.20058 PMC8133879 33226327

[B28] Garcia D. Erkan D. (2018). Diagnosis and management of the antiphospholipid syndrome. N. Engl. J. Med. 378 (21), 2010–2021. 10.1056/NEJMra1705454 29791828

[B29] Ghembaza A. Saadoun D. (2020). Management of antiphospholipid syndrome. Biomedicines 8 (11), E508. 10.3390/biomedicines8110508 33212808 PMC7696303

[B30] Giannakopoulos B. Krilis S. A. (2013). The pathogenesis of the antiphospholipid syndrome. N. Engl. J. Med. 368 (11), 1033–1044. 10.1056/NEJMra1112830 23484830

[B31] Hughes G. R. (1983). Thrombosis, abortion, cerebral disease, and the lupus anticoagulant. Br. Med. J. 287 (6399), 1088–1089. 10.1136/bmj.287.6399.1088 6414579 PMC1549319

[B32] Karahan S. Erol K. Yuksel R. C. Artan C. Celik I. (2022). Antiphospholipid antibodies in COVID-19-associated pneumonia patients in intensive care unit. Mod. Rheumatol. 32 (1), 163–168. 10.1080/14397595.2021.1892257 33620009 PMC9383176

[B33] Koval N. Alves M. Plácido R. Almeida A. G. Fonseca J. E. Ferreira J. J. (2021). Direct oral anticoagulants versus vitamin K antagonists in patients with antiphospholipid syndrome: Systematic review and meta-analysis. RMD Open 7 (2), e001678. 10.1136/rmdopen-2021-001678 34253684 PMC8276293

[B34] Kravvariti E. Koutsogianni A. Samoli E. Sfikakis P. P. Tektonidou M. G. (2020). The effect of hydroxychloroquine on thrombosis prevention and antiphospholipid antibody levels in primary antiphospholipid syndrome: A pilot open label randomized prospective study. Autoimmun. Rev. 19 (4), 102491. 10.1016/j.autrev.2020.102491 32084592

[B35] Lin W. Luo Y. Liu F. Li H. Wang Q. Dong Z. (2022). Status and trends of the association between diabetic nephropathy and diabetic retinopathy from 2000 to 2021: Bibliometric and visual analysis. Front. Pharmacol. 13, 937759. 10.3389/fphar.2022.937759 35795563 PMC9251414

[B36] Liu Y. Zheng B. Hong J. Liu Y. (2022). Mapping theme trends and knowledge structure of sjögren’s syndrome (SS), A bibliometric analysis from 2010 to 2021. Clin. Rheumatol. 41 (9), 2779–2789. 10.1007/s10067-022-06196-x 35567664

[B37] Luigi Meroni P. Toubi E. Shoenfeld Y. (2019). Are anti-phospholipid syndrome and systemic lupus erythematosus two different diseases? A 10-year late remake. Isr. Med. Assoc. J. 21 (7), 491–493. 31507127

[B38] Ma D. Yang B. Guan B. Song L. Liu Q. Fan Y. (2021). A bibliometric analysis of pyroptosis from 2001 to 2021. Front. Immunol. 12, 731933. 10.3389/fimmu.2021.731933 34484243 PMC8416445

[B39] Mazetto B. de M. Hounkpe B. W. da Silva Saraiva S. Vieira-Damiani G. Dos Santos A. P. R. Jacinto B. C. (2022). Association between neutrophil extracellular traps (NETs) and thrombosis in antiphospholipid syndrome. Thromb. Res. 214, 132–137. 10.1016/j.thromres.2022.05.001 35561448

[B40] Meng H. Yalavarthi S. Kanthi Y. Mazza L. F. Elfline M. A. Luke C. E. (2017). *In vivo* role of neutrophil extracellular traps in antiphospholipid antibody-mediated venous thrombosis. Arthritis Rheumatol. 69 (3), 655–667. 10.1002/art.39938 27696751 PMC5329054

[B41] Miyakis S. Lockshin M. D. Atsumi T. Branch D. W. Brey R. L. Cervera R. (2006). International consensus statement on an update of the classification criteria for definite antiphospholipid syndrome (APS). J. Thromb. Haemost. 4 (2), 295–306. 10.1111/j.1538-7836.2006.01753.x 16420554

[B42] Nuri E. Taraborelli M. Andreoli L. Tonello M. Gerosa M. Calligaro A. (2017). Long-term use of hydroxychloroquine reduces antiphospholipid antibodies levels in patients with primary antiphospholipid syndrome. Immunol. Res. 65 (1), 17–24. 10.1007/s12026-016-8812-z 27406736

[B43] Ordi-Ros J. Saez-Comet L. Perez-Conesa M. Vidal X. Riera-Mestre A. Castro-Salomo A. (2019). Rivaroxaban versus vitamin K antagonist in antiphospholipid syndrome: A randomized noninferiority trial. Ann. Intern. Med. 171 (10), 685–694. 10.7326/M19-0291 31610549

[B44] Pengo V. Denas G. Zoppellaro G. Jose S. P. Hoxha A. Ruffatti A. (2018). Rivaroxaban vs warfarin in high-risk patients with antiphospholipid syndrome. Blood 132 (13), 1365–1371. 10.1182/blood-2018-04-848333 30002145

[B45] Pericleous C. Ferreira I. Borghi O. Pregnolato F. McDonnell T. Garza-Garcia A. (2016). Measuring IgA anti-β2-glycoprotein I and IgG/IgA anti-domain I antibodies adds value to current serological assays for the antiphospholipid syndrome. PLoS One 11 (6), e0156407. 10.1371/journal.pone.0156407 27253369 PMC4890741

[B46] Petri M. (2020). Antiphospholipid syndrome. Transl. Res. 225, 70–81. 10.1016/j.trsl.2020.04.006 32413497 PMC7487027

[B47] Petri M. (2011). Use of hydroxychloroquine to prevent thrombosis in systemic lupus erythematosus and in antiphospholipid antibody-positive patients. Curr. Rheumatol. Rep. 13 (1), 77–80. 10.1007/s11926-010-0141-y 20978875

[B48] Reshetnyak T. Cheldieva F. Cherkasova M. Lila A. Nasonov E. (2022). IgA antiphospholipid antibodies in antiphospholipid syndrome and systemic lupus erythematosus. Int. J. Mol. Sci. 23 (16), 9432. 10.3390/ijms23169432 36012697 PMC9409442

[B49] Rodriguez-Pinto I. Espinosa G. Erkan D. Shoenfeld Y. Cervera R. CAPS Registry Project Group (2018). The effect of triple therapy on the mortality of catastrophic anti-phospholipid syndrome patients. Rheumatology 57 (7), 1264–1270. 10.1093/rheumatology/key082 29660074

[B50] Rodriguez-Pinto I. Moitinho M. Santacreu I. Shoenfeld Y. Erkan D. Espinosa G. (2016). Catastrophic antiphospholipid syndrome (CAPS): Descriptive analysis of 500 patients from the international CAPS registry. Autoimmun. Rev. 15 (12), 1120–1124. 10.1016/j.autrev.2016.09.010 27639837

[B51] Ruiz-Irastorza G. Crowther M. Branch W. Khamashta M. A. (2010). Antiphospholipid syndrome. Lancet 376 (9751), 1498–1509. 10.1016/S0140-6736(10)60709-X 20822807

[B52] Ruiz-Irastorza G. Cuadrado M. J. Ruiz-Arruza I. Brey R. Crowther M. Derksen R. (2011). Evidence-based recommendations for the prevention and long-term management of thrombosis in antiphospholipid antibody-positive patients: Report of a task force at the 13th international congress on antiphospholipid antibodies. Lupus 20 (2), 206–218. 10.1177/0961203310395803 21303837

[B53] Schreiber K. Sciascia S. de Groot P. G. Devreese K. Jacobsen S. Ruiz-Irastorza G. (2018). Antiphospholipid syndrome. Nat. Rev. Dis. Prim. 4, 17103. 10.1038/nrdp.2017.103 29321641

[B54] Sciascia S. Amigo M. C. Roccatello D. Khamashta M. (2017). Diagnosing antiphospholipid syndrome: ‘Extra-Criteria’ manifestations and technical advances. Nat. Rev. Rheumatol. 13 (9), 548–560. 10.1038/nrrheum.2017.124 28769114

[B55] Sciascia S. Sanna G. Murru V. Roccatello D. Khamashta M. A. Bertolaccini M. L. (2013). Gapss: The global anti-phospholipid syndrome score. Rheumatology 52 (8), 1397–1403. 10.1093/rheumatology/kes388 23315788

[B56] Sciascia S. Sanna G. Murru V. Roccatello D. Khamashta M. A. Bertolaccini M. L. (2015). The global anti-phospholipid syndrome score in primary APS. Rheumatology 54 (1), 134–138. 10.1093/rheumatology/keu307 25122726

[B57] Shen J. Shen H. Ke L. Chen J. Dang X. Liu B. (2022). Knowledge mapping of immunotherapy for hepatocellular carcinoma: A bibliometric study. Front. Immunol. 13, 815575. 10.3389/fimmu.2022.815575 35173728 PMC8841606

[B58] Tebo A. E. Willis R. Jaskowski T. D. Guerra M. Pierangeli S. S. Salmon J. (2016). Clinical significance and correlations between anti-β2 glycoprotein I IgA assays in antiphospholipid syndrome and/or systemic lupus erythematosus. Clin. Chim. Acta. 460, 107–113. 10.1016/j.cca.2016.06.025 27346478

[B59] Tektonidou M. G. Andreoli L. Limper M. Amoura Z. Cervera R. Costedoat-Chalumeau N. (2019). EULAR recommendations for the management of antiphospholipid syndrome in adults. Ann. Rheum. Dis. 78 (10), 1296–1304. 10.1136/annrheumdis-2019-215213 31092409 PMC11034817

[B60] Trahtemberg U. Rottapel R. Dos Santos C. C. Slutsky A. S. Baker A. Fritzler M. J. (2021). Anticardiolipin and other antiphospholipid antibodies in critically ill COVID-19 positive and negative patients. Ann. Rheum. Dis. 80 (9), 1236–1240. 10.1136/annrheumdis-2021-220206 33903092 PMC8076626

[B61] van Eck N. J. Waltman L. (2010). Software survey: VOSviewer, a computer program for bibliometric mapping. Scientometrics 84 (2), 523–538. 10.1007/s11192-009-0146-3 20585380 PMC2883932

[B62] Wiggins B. S. Dixon D. L. Neyens R. R. Page R. L. Gluckman T. J. (2020). Select drug-drug interactions with direct oral anticoagulants: JACC review topic of the week. J. Am. Coll. Cardiol. 75 (11), 1341–1350. 10.1016/j.jacc.2019.12.068 32192661

[B63] Wilson W. A. Gharavi A. E. Koike T. Lockshin M. D. Branch D. W. Piette J. C. (1999). International consensus statement on preliminary classification criteria for definite antiphospholipid syndrome: Report of an international workshop. Arthritis Rheum. 42 (7), 1309–1311. 10.1002/1529-0131(199907)42:7<1309::AID-ANR1>3.0.CO;2-F 10403256

[B64] Wu H. Li Y. Tong L. Wang Y. Sun Z. (2021). Worldwide research tendency and hotspots on hip fracture: A 20-year bibliometric analysis. Arch. Osteoporos. 16 (1), 73. 10.1007/s11657-021-00929-2 33866438

[B65] Zhang Y. Xiao M. Zhang S. Xia P. Cao W. Jiang W. (2020). Coagulopathy and antiphospholipid antibodies in patients with COVID-19. N. Engl. J. Med. 382 (17), e38. 10.1056/NEJMc2007575 32268022 PMC7161262

[B66] Zhu S. Liu Y. Gu Z. Zhao Y. (2021). A bibliometric analysis of advanced healthcare materials: Research trends of biomaterials in healthcare application. Adv. Healthc. Mat. 10 (10), e2002222. 10.1002/adhm.202002222 33599117

